# Reverse electrical remodeling in rats with heart failure and preserved ejection fraction

**DOI:** 10.1172/jci.insight.173086

**Published:** 2023-07-10

**Authors:** Jae Hyung Cho, Peter J. Kilfoil, Rui Zhang, Ryan E. Solymani, Catherine Bresee, Elliot M. Kang, Kristin Luther, Russell G. Rogers, Geoffrey de Couto, Joshua I. Goldhaber, Eduardo Marbán, Eugenio Cingolani

Original citation *JCI Insight*. 2018;3(19):e121123. https://doi.org/10.1172/jci.insight.121123

Citation for this retraction: *JCI Insight*. 2023;8(13):e173086. https://doi.org/10.1172/jci.insight.173086

The Smidt Heart Institute of Cedars-Sinai Medical Center recently notified *JCI Insight* of data misrepresentation in this article, discovered in the process of internal review. In particular, natural history controls without surgical intervention were admixed with data from rats randomized to receive surgical placebo infusion, without correct attribution. The authors have indicated that the conclusions of the article may be valid; however, the corresponding author and the institution deemed the data misrepresentation sufficiently substantive to request retraction. Therefore, *JCI Insight* is retracting this article.

## Version 1 07/10/2023

Electronic publication

